# A short review on the root canal configuration of primary maxillary molars

**DOI:** 10.6026/973206300161033

**Published:** 2020-12-31

**Authors:** Mahesh Ramakrishnan, MS Niveditha, Deepa Gurunathan

**Affiliations:** 1 Saveetha Dental College, Saveetha Institute of Medical and Technical Sciences, Saveetha University, Chennai 77, India

**Keywords:** CBCT, pediatric endodontics, primary molar, root canal configuration

## Abstract

It is of interest to compile available information on the root canal morphology of primary maxillary molars from known literature. The literature resources used to collect data include Medline/PubMed, The Cochrane Central Register of Clinical Trials, SIGLE and Science Direct. Data consists of type of population, number of teeth per study, number of root canals, canal length and type of root canal configuration. We used data from a total of 13 studies (951 primary maxillary molars). Maxillary molars (1st and 2nd) are dominant for two roots variant. The first molar the mean root length ranges from 7.9mm - 8.1mm. The second molar ranges from 7.2mm-8.5mm. Type I (explain in a phrase) canal morphology is the common variant in both the molars. Data shows that Root Canal morphology shows variations with the diagnostic aid (example micro CT) used and in different ethnic populations.

## Background

There is an increase in Endodontic therapy procedures in primary molars rather than extraction, since it is the best space maintainer. The need to maintain the primary teeth in the occlusion until exfoliation and eruption of permanent teeth is desirable, since it acts as an ideal space maintainer [1]. Understanding the canal configuration of primary molars plays a vital role the success of any endodontic therapy. Advances in radiological examination such as with the introduction of micro CT imaging techniques for detailed study of tooth anatomy, there is an increase understanding the external and internal anatomy of the tooth structure, canal volume and accessory canals. It is of interest to compile available information on the root canal morphology of primary maxillary Long term clinical prognosis depends upon identification of the complexity of the canal and complete debridement. Complete canal disinfection with irrigation and intra canal medicaments are impossible if the clinician is not aware of various canal morphological variations. Canal variations also show great difference with ethnic population and with various diagnostic aids. Updating our knowledge from laboratory studies and use of advanced diagnostic aids is essential to provide insight into the complex root canal anatomy. Primary maxillary molars continue to present a unique challenge to pediatric dentist because of the tortuous and bizarre morphology of their root canal. In Maxillary molars, the double root variant in which fusion between both the disto buccal and palatal roots is the predominant type, in first molar it ranges from 60-77% and this percentage is lesser in second molar 22.5%. Three roots and three canals is the most common canal morphology in both the first and second maxillary molar [2]. Maxillary fist molar the mean root length ranges from 7.9mm - 8.1mm in the mesio buccal root, 6.7-7.3mm in disto buccal and 5.9mm-7.7mm in palatal root. In Second molar mesio buccal root length ranges from7.2mm-8.5mm, disto buccal 6.5mm-8.06mm, palatal root ranges from 7.4mm-9.92mm. The volume of the canal in 3dimensional structure can be evaluated. The study done by fumes 3 estimated the volume in first molar to be 2.8mm3 in mesio buccal root, disto buccal 1.3mm3 and palatal root 2.9mm3. In the second molar the Palatal root had the more volume 5.4mm3 mesio buccal roots was 3.2mm3 disto buccal 1.0mm3. In the first molar Type I canal configuration was more prevalent in mesio buccal (88.9%-93.10%), disto buccal (95.65%-100%) and palatal (100%). In the second molar Mesio buccal root had Type I canal configuration in 90% of teeth and type IV in 10% teeth. Disto buccal had Type I in 100% of the teeth [3-8]. In maxillary molars the mesio buccal root had maximum angulations (18.66°), followed by the disto buccal root (15.40°), palatal root (12.29°) showed the least angulations. In the second molar the palatal root showed the maximum angulations (16.14°), followed by the mesio buccal root (10.71°), disto buccal root (8.78°) showed the least angulations [9-13].

## Conclusion:

Available data shows that root canal morphology shows variations with the diagnostic aid (example micro CT) used and in different ethnic populations.
